# A case of hypertrophic cardiomyopathy with previous aortic valve replacement

**DOI:** 10.1186/s13019-024-02608-y

**Published:** 2024-03-19

**Authors:** Hongyan Xiao, Laichun Song, Meng Guo, Liang Tao

**Affiliations:** 1 Department of Cardiac Surgery, Wuhan Asia Heart Hospital, Wuhan University of Science and Technology, Wuhan, 430022 Hubei P.R. China; 2 Wuhan Clinical Research Center for Cardiomyopathy, Wuhan, Hubei P.R. China

**Keywords:** Case report, Hypertrophic cardiomyopathy, Percutaneous intra-myocardial septal radiofrequency ablation

## Abstract

We describe a 45-year-old patient who was diagnosed with hypertrophic obstructive cardiomyopathy (HOCM) after the aortic valve replacement surgery. Enlarged left atria, thickened ventricular septum, left ventricular outflow tract stenosis, moderate mitral regurgitation and mild tricuspid regurgitation in the echocardiography were found. We offered the patient the new minimally invasive treatment modality: percutaneous intra-myocardial septal radiofrequency ablation (PIMSRA). We demonstrate the safety and efficacy with pictures. One month after surgery, the patient recovered well with improved symptoms of chest tightness, and no LVOT obstruction or arrhythmia.

## Introduction

**Fig. 1:**
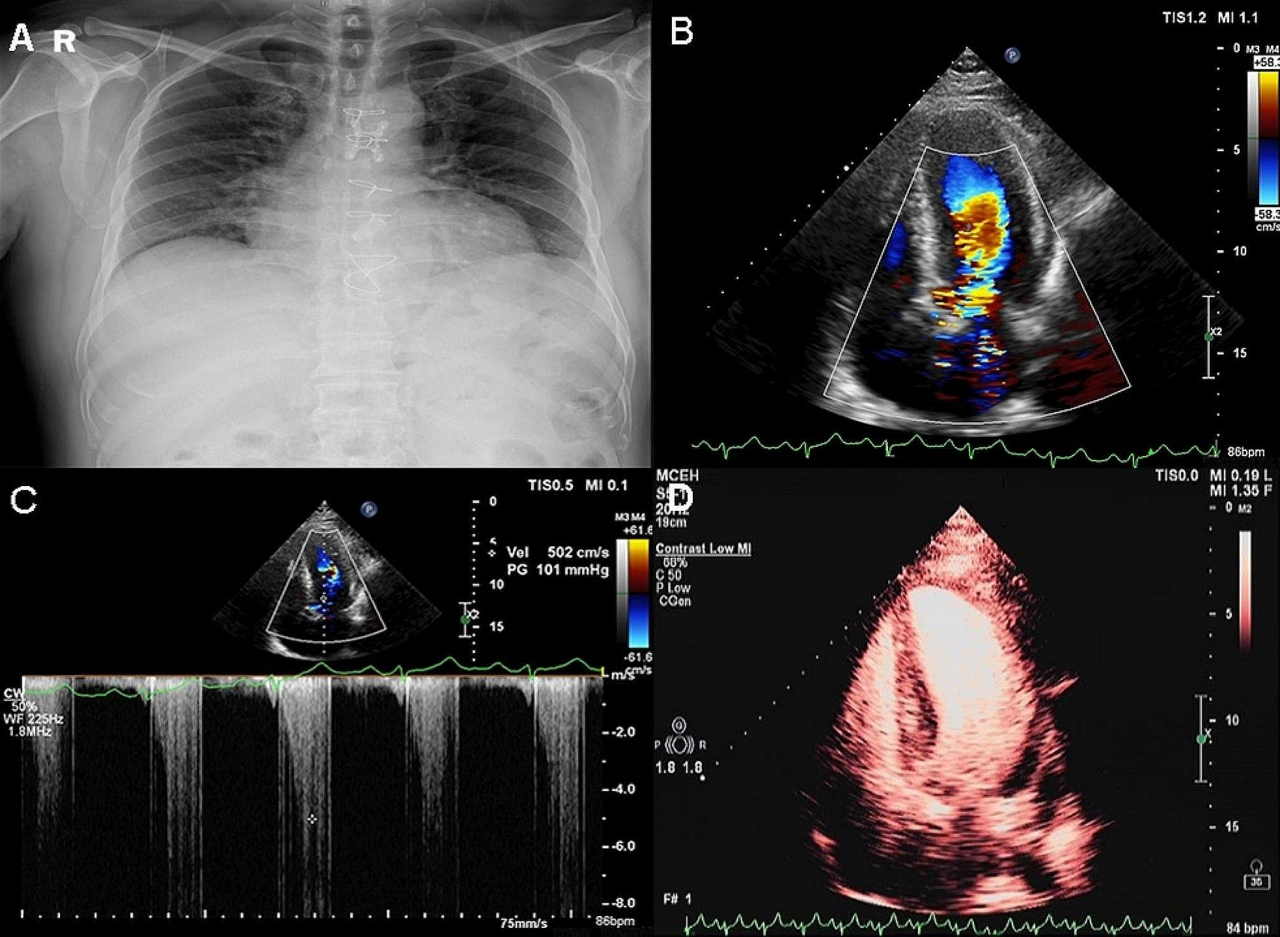
**A**: Chest X-ray; **B**: Color Doppler flow.; **C**: The systolic pressure gradient of the LVOT. **D**: Left ventricular acoustic contrast

A 45-year-old man presented with body weakness and dizziness. The patient underwent a successful aortic valve replacement (AVR) surgery using a mechanical valve on May 19, 2022 (not performed in our center). However, the patient with oral metoprolol 47.5 mg/day after AVR, heart rate maintained at around 60 beats, still exhibited chest tightness and wheezing after activity in the next 6 months. He was eventually referred to our Hypertrophic Cardiomyopathy Center for further evaluation. The patient was obese (height: 168 cm, weight: 95 kg, and body mass index (BMI): 33.7 kg/m^2^). Chest X-ray showed no lung consolidation and slightly larger heart shadow (Fig. 1A). Enlarged left atria, thickened ventricular septum, left ventricular outflow tract stenosis, and moderate mitral regurgitation and mild tricuspid regurgitation in the echocardiography were found (Fig. 1B). Two-dimensional transthoracic echocardiography (TTE) measured a pressure gradient in the LVOT of 101 mmHg (peak); the thickest region of interventricular septum (IVS) to be 28 mm; mitral systolic anterior motion (SAM motion) (Fig. 1C). Left ventricular acoustic contrast showed uneven hypertrophy of ventricular septum and left ventricular wall (Fig. 1D).

Treatment options for this patient were discussed extensively, including surgical myectomy and alcoholic ventricular septal ablation (ASA). Considering the patient’s cardiac status and his desire not to undergo thoracotomy, we offered the patient the new minimally invasive treatment modality: percutaneous intra-myocardial septal radiofrequency ablation (PIMSRA). The Institutional Ethics Committee (2019-YXXJS-017) of Wuhan Asia Heart Hospital Hospital approved the procedure, which was performed in accordance with the ethical standards of the Declaration of Helsinki. Written consent was obtained.

**Fig. 2:**
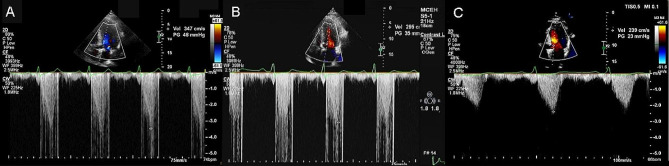
**A**: LVOT gradient of 48 mm Hg after the surgery; **B**: LVOT gradient of 35 mm Hg 1 month after the PIMSRA surgery. **C**: LVOT gradient of 23 mm Hg 6 month after the PIMSRA surgery

The patient was placed in the left decubitus position (Right shoulder raised 30 degrees) and intubated with general anesthesia. Under the guidance of echocardiography, the Cool-Tip ACT-2020 radiofrequency needle (17G, Cool-tip™ RF Ablation System and Switching Controller, Medtronic Minimally Invasive Therapies, Minneapolis, MN, United States) was inserted through the apex of the heart along the long axis of the IVS into the hypertrophic area of the anterior IVS. The ablation energy of 70 W was applied for 12 min. Real-time ultrasonic monitoring showed that the ablation area began to vaporize from the tip of the needle, and the gasification range was gradually expanded, and the echo of the treatment area was significantly enhanced. After the ablation machine appeared hibernation, the needle was removed to the middle and posterior of the anterior interval, and the ablation was continued until hibernation by the same method. We achieved the following results at the end of the procedure: LVOT gradient: 48 mm Hg at rest (Fig. 2A). Postoperative IVS thickness did not change from preoperative IVS. At his follow-up in 1 month and 3 month, LVOT gradient was reduced to 35 mm Hg and 23 mm Hg, respectively (Fig. 2B, C). Decrease in IVS to 20 mm occurred gradually after the procedure over 1 and 6 months (Fig. 2B, C). No temporary pacemaker was placed during this patient’s PIMSRA procedure. No atrioventricular block or bundle branch block was observed postoperatively. No complications occurred, and his symptoms resolved.

## Discussion

Patients with hypertrophic cardiomyopathy (HCM) combined with left ventricular outflow tract obstruction (LVOTO) often present with severe symptoms and functional limitations. Remission of LVOTO can be achieved with two invasive interventions, surgical myectomy and alcohol septal ablation (ASA). Surgical myectomy involves sternotomy, which requires high tolerance and prolonged hospitalization. ASA is a less invasive approach, but the variability of the arterial anatomy and collateral recruitment of septal branches may lead to the misplacement of alcohol clots. A common complication of myectomy and ASA is impairment of the conduction system. The 2011 American College of Cardiology Foundation/American Heart Association Guidelines state that surgical myomectomy is the gold standard of medical treatment for patients with refractory obstructive HCM and that ASA should be reserved for elderly patients or those with severe comorbidities [1]. In contrast to surgical myotomy and alcoholic septal ablation (ASA), PIMSRA uses ultrasound guidance to access the left ventricular apex directly, avoiding coronary artery and conduction system injury. In a study by Zhou et al. of 200 HOCM patients who underwent PIMSRA, IVS and left ventricular outflow tract gradient were both decreased at follow-up [2]. Thirty-day major adverse clinical events rate and other periprocedural complication rates were relatively low [2]. A previous study of 11 HOCM patients conducted by our centre found similar results [3]. IVS and LOVT were significantly decreased compared with those in pre-operation [3]. These findings suggest that PIMSRA may be an effective way to relieve symptoms of LVOTO and acceptable complication rates in patients with drug-refractory HOCM. Because the anatomical status of the patient’s heart in this study did not support ASA surgery, we finally used PIMSRA to provide an effective alternative for patients previously undergoing mechanical aortic valve replacement to alleviate severe LVOT obstruction. Patients will continue to be followed up later with a view to having provided an effective alternative for other inoperable patients.

## Data Availability

All data generated or analyzed during this study are included in this published article.
